# Infrared thermography for non-invasive measurement of social inequality aversion in rodents and potential usefulness for future animal-friendly studies

**DOI:** 10.3389/fnbeh.2023.1131427

**Published:** 2023-03-06

**Authors:** Shigeru Watanabe

**Affiliations:** Department of Psychology, Keio University, Minato, Japan

**Keywords:** infrared thermography, stress-induced hyperthermia, inequality aversion, advantageous inequality, disadvantageous inequality

## Abstract

Infrared thermography is a method that detects thermal radiation energy and can measure the body surface temperature of animals from a distance. While rectal temperature has traditionally been used to measure animals’ core temperature, thermal imaging can avoid the stress and potential rise of body temperature deriving from handling of the animals. Additionally, being non-invasive and contactless, thermal imaging allows free movement of the animals. The validity of this technique as a psychophysiological method has been proven in a series of stress-induced hyperthermia (SIH) studies of mice under social inequality conditions. Restraint in a holder elicits SIH in mice. A restrained mouse surrounded by freely moving cage mates displays increased SIH suggesting that social inequality enhances the stress. Social inequality can be examined also in unrestrained mice, in particular through unequal distribution of food. In this protocol, a food-deprived mouse is given a small piece of cheese, while its cage mate is given a large piece of cheese. This inequity causes SIH, suggesting social inequality aversion in mice. Thus, social inequality in different situations similarly increased SIH. Importantly, in future studies infrared thermography could also be used to evaluate emotional arousal states different from stress (for example to assess reactivity to rewards or in social and sexual preference tests). Moreover, the technique could be used to investigate also cognitive arousal induced by novelty. Indeed, infrared thermography could be a particularly useful tool for animal-friendly studies of cognition and emotion in rodents.

## 1. The mechanism of infrared thermography

Infrared radiation is emitted naturally from any object with a temperature higher than zero. The relationship between the infrared radiation and the surface temperature of an object is expressed by the Stephen–Boltzmann formula, using an ideal object called a black body. The radiation ratio of a black body is equal to 1.0; however, when infrared thermography is performed on an animal, the radiation ratio of its body surface is estimated by matching the temperature recorded by an infrared thermometer to that obtained by a contact thermometer. Importantly, infrared thermography is non-invasive and contactless, allowing the animals to move freely without any disturbance during temperature measurement, which makes this method completely animal-friendly and particularly suitable for behavioral research. In the case of rodents, the temperature of the interscapular region is commonly used for measurement purposes. To obtain accurate measurements, it is best to remove the hair of the animals by shaving or use nude mice, as, although hair does not produce heat, it can maintain it (Fiebig et al., 2018). Since radiation travels in straight lines, measuring the intensity of thermal radiation using an acute angle reduces the radiation received. Therefore, although continuous long-term thermographic recording is feasible and can be applied to freely moving wild animals (Vinne et al., 2020), this angle dependency may result in data variability.

## 2. Analysis of social inequality aversion by stress-induced hyperthermia

Stress causes several autonomic responses, including changes in the heart rate, blood pressure, and respiration rate. Stress also increases body temperature, a phenomenon known as stress-induced hyperthermia (SIH; Bouwknecht et al., 2007). A variety of stressors induce hyperthermia, including being in a novel cage (Houtepen et al., 2011), physical restraint (Thornhill et al., 1979; Van der Heyden et al., 1997; Van Eijl et al., 2006), social threat (Keeney et al., 2001; Pardon et al., 2004), and fear conditioning (Marks et al., 2009). The rectal temperature has traditionally been used to measure the core temperature of animals (e.g., Van der Heyden et al., 1997); however, infrared thermography has been employed as a non-invasive alternative (Conley and Hutson, 2007; Hishimura and Itoh, 2009; Houtepen et al., 2011). Compared to rectal temperature measurement, thermal imaging can avoid the stress and the consequent potential rise of body temperature deriving from handling of the animals. Although stress induction is, by definition, not stress-free, it is important to employ stress-free methods for the experimental measurements. On the one hand, this represents a refinement of the experimental procedure, increasing animal welfare. On the other hand, when studying stress processes it is particularly useful to choose a measurement method that does not interfere with the variable of interest.

According to Nakamura (2015), the central mechanisms of SIH are as follows. Psychological stress activates two groups of neurons in the dorsomedial hypothalamus (DMH): the dorsal DMH neurons and the ventral DMH neurons. Dorsal DMH neurons send glutamatergic input to sympathetic premotor neurons in the rostral medullary raphe to drive brown adipose tissue (BAT) thermogenesis, whereas ventral DMH neurons send direct input to the paraventricular hypothalamus to activate the hypothalamus-pituitary-adrenal axis, releasing stress hormones. BAT is a specialized organ for rapid heat production. In mice, it is found mainly in the interscapular region, an area highly innervated by the sympathetic nerves (Robinson et al., 2016).

Body temperature commonly shows individual variations and is easily affected by environmental changes such as being in a novel experimental setting. To account for this, instead of using the absolute temperature as a dependent variable, the difference between the temperature at baseline and the temperature in the experimental condition can be used. Additionally, it is important to make sure that animals are well adapted.

## 3. Social inequality aversion in rodents

Humans seek to punish unfair behavior of others (Fehr and Gachter, 2002), indicating strong inequality aversion in this species. Inequality aversion has also been observed in primates (Brosnan and de Waal, 2014) and, to a certain extent, in dogs (Range et al., 2008, 2012). However, there have been many challenges in identifying social inequality aversion in other species (Oberliessen and Kalenscher, 2019) and there still are contradictory discussions on the subject. An important topic of discussion is the method used to measure aversion. Since aversion leads to the behavioral avoidance of its source and induces physiological stress, it can be measured both behaviorally and physiologically. The author has employed infrared thermography to measure aversion as an autonomic response in mice and obtained consistent results, which will be reviewed in the following paragraphs.

### 3.1. Social inequality in restraint stress

**Figure 1:**
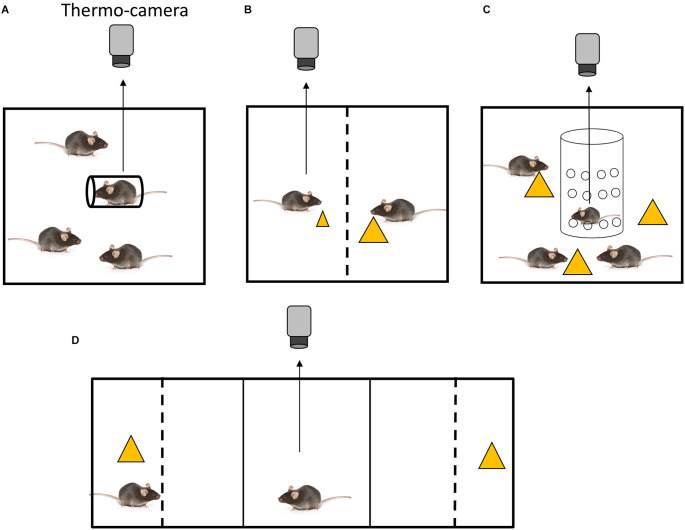
Experimental settings for the assessment of social inequality aversion in mice. **(A)** Setting to test inequality aversion to restraint stress (Watanabe, 2015). A mouse holder for blood sampling (diameter, 3 cm; length, 10 cm) was used to restrain the mouse. All animals were placed in cages (20 × 15 × 13 cm). **(B)** Setting to test inequality aversion in food distribution (Watanabe, 2017). An arena made of transparent acrylic plastic was employed. A central partition, a transparent acrylic plastic barrier with small holes (diameter: 0.5 cm; distance between holes: 0.3 cm), divided the arena into two equally sized chambers. Each chamber measured 19 × 11 × 20 cm. One mouse received a small piece of cheese, while the other one received a large piece. **(C)** Setting to test disadvantageous inequality aversion in food distribution (Watanabe, 2019). The experimental chamber was a 20 × 25 × 20 cm acrylic box. A transparent cylindrical tube (diameter, 10 cm; height, 20 cm) made of acrylic and featuring several holes (diameter of 0.5 cm with a distance of 0.3 cm between each hole) was placed vertically in the center of the experimental chamber. The test mouse was then placed in the cylinder. Yellow triangles indicate pieces of cheese. **(D)** Setting for the simultaneous recording of behavioral preference and body temperature (Watanabe, 2017). The apparatus used for the behavioral tests was a conventional conditioned place preference apparatus (MED ENV3015) with three compartments: two lateral compartments (16 × 13 × 12 cm) and a central compartment (6 × 13 × 12 cm). The central compartment was connected to the two lateral compartments through guillotine doors. A box made of gray acrylic plate was placed in each lateral compartment, so that the external appearance of the lateral compartments was identical. In each box, a transparent acrylic partition was placed 5 cm from the end wall to create a separated stimulus area. In this setting preference between a cage mate eating cheese (left) and a piece of cheese without cage mate (right) was tested. The subject mouse was placed in the center.

Placing animals in cylindrical holders induces restraint stress. Mice that were restrained in holders alone in the presence of freely moving cage mates (the social inequality condition) have been shown to exhibit a greater degree of SIH (Watanabe, 2015) compared to those that were restrained in the presence of other equally restrained cage mates (the social equality condition). The outcome of the first condition is indicative of social inequality aversion (Figure 1A), whereas the outcome of the second condition is indicative of social buffering.

Stress has a memory-enhancing effect on aversive experiences (Hashimoto and Watanabe, 2005; Miracle et al., 2006; Roozendaal et al., 2009), and Watanabe (2011) investigated this effect in mice under three social conditions. The experience of restraint stress enhanced the aversive memory of a floor that delivered an electric shock (the single stress condition). The aversive memory enhancement was reduced in mice that were restrained in the presence of similarly restrained cage mates and increased in the presence of freely moving cage mates. Corticosterone levels, which are a common biomarker of stress, were highest after restraint stress was applied with freely moving cage mates, and lowest after restraint stress was applied with restrained cage mates. These results are consistent with those of the aforementioned thermography experiment, which demonstrated that social inequality enhances SIH (Watanabe, 2015). This stress enhancement could be explained by the possibility of predation. Indeed, the social equality condition has a dilution effect against predation, whereas a restrained animal among freely moving conspecifics could easily be a target of predation.

Social inequality is divided into two types: disadvantageous and advantageous. For humans, both types of inequality have aversive properties (Fehr and Schmidt, 1999). A restrained mouse surrounded by freely moving cage mates experiences disadvantageous inequality, whereas a free mouse surrounded by restrained cage mates experiences advantageous inequality. The body temperature of a freely moving mouse in the presence of restrained cage mates has been previously investigated, but no clear increase in temperature has been observed. Therefore, the existence of aversion to advantageous inequality in mice is questionable (Watanabe, 2019). A preference test between a compartment with a freely moving cage mate and that with a restrained mate did not show signs of avoidance of the condition with the restrained mate (Watanabe, 2012), but using a conditioned place conditioning protocol, the freely moving mice showed place aversion for the chamber where the mate was restrained (Watanabe, 2012), indicating that a certain degree of advantageous inequality aversion could be present in mice.

### 3.2. Social inequality in food delivery

Restraint stress is a well-established method for inducing stress in rodents, but it implies a high level of physical restriction. Alternative stress induction protocols allow analysis of social inequality aversion in freely moving mice. For instance, the aversive property of inequitable food delivery has been demonstrated in several non-human primates (Yamamoto and Takimoto, 2012; Brosnan and de Waal, 2014; see Bräuer et al., 2009, and Sheskin et al., 2014 for contradictory discussion). The body temperature of mice has been examined under social equality and inequality conditions of food delivery in a test chamber with two compartments, each containing a mouse (Watanabe, 2017). In the equality condition, the same amount of food (cheese) was provided to two similarly food-deprived mice. However, in the inequality condition, different amounts of food were provided to the two food-deprived mice (Figure 1B). An increase in body temperature was observed in mice that were given a small piece of cheese while their cage mate received a large piece. Thus, this finding indicates that social inequality in food delivery leads to SIH. Interestingly, when one mouse was given laboratory chew, which is considered to be less preferable, and the other was given cheese, which is considered to be more preferable, the former did not exhibit SIH. Hence, mice appear to be sensitive to quantitative, but not qualitative, inequality. On the contrary, capuchin monkeys showed inequality aversion when they received a cucumber (non-preferred food) while their counterparts received grapes (preferred food; Brosnan and de Waal, 2014). However, because the relative value of these foods might differ between mice and capuchin monkeys, it is premature to conclude that qualitative inequality aversion does not occur in mice.

Analogously to the restraint experiment, when a previously food-deprived mouse is tested in a situation in which it does not receive food and it is surrounded by cage mates that are consuming food (disadvantageous inequality condition), it has been shown to exhibit SIH (Watanabe, 2019; Figure 1C). When a mouse receives food while being surrounded by food-deprived cage mates that do not have access to food (advantageous inequality condition), an increase in body temperature has been observed, although it was not significantly higher than that found in the equality condition. This increase could be explained by the fact that the eating mouse was confined in a smaller area of the arena. The freely moving cage mates could not physically access the eating mouse, which was confined in an area delimited by an acrylic cylinder, but they could gather around the acrylic cylinder wherein the subject was eating cheese. This behavior of the freely moving cage mates might have caused in the eating mouse a low stress deriving from the risk of food pilferage. This behavior of the freely moving cage mates might have caused in the eating mouse a low stress deriving from confinement, which could explain the small increase (not significant) in body temperature. In summary, disadvantageous inequality aversion was observed in mice in the presence of both unpleasant and pleasant stimuli (restraint and food, respectively), while advantageous inequality aversion was not. Notably, pre-feeding of the test mouse has been shown to attenuate SIH under disadvantageous conditions (Watanabe, 2019). Therefore, the sight of cage mates that are eating does not seem to have an aversive effect on pre-fed mice with limited access to food. These findings indicate that, when a mouse is exposed to conspecifics that are eating while its access to food is limited, it may perceive the situation as a potential depletion of food, which leads to an aversive effect. However, pre-feeding the mouse reduces this aversive effect.

Additionally, Oberliessen et al. (2016) examined inequality aversion in rats using a T-maze choice paradigm, which showed a preference for equal food delivery compared to unequal delivery. Thus, the rats involved in this study also showed inequality aversion.

## 4. Contradiction between behavioral preference and autonomic response

Thorndike defined satisfaction as “that animal does not nothing to do avoid, often doing something which maintains or renews it, and the annoying state as that animal does nothing to preserve, often doing something which puts an end to it“ (Thorndike, 1911; page 2). Behavioral aversion to social inequality is measured as the time spent in two compartments (Watanabe, 2017). In an experiment performed using a two-choice apparatus, the test mice were able to observe the content of two compartments, one containing a cage mate eating cheese and one containing cheese alone (Figure 1D). Due to the presence of the partition, the subjects could not physically access the cage mate or cheese. Test mice spent more time in a compartment with a cheese-eating cage mate than in a compartment that included either cheese alone or a cage mate alone. This finding suggests that disadvantageous inequality may have a “satisfactory effect” in the sense of Thorndike’s definition.

In a second experiment performed in the same apparatus, behavioral preferences and body temperatures were simultaneously recorded. As in the previous experiment, mice spent more time in the unequal condition compartment (observing a cheese-eating cage mate) and an increase in body temperature was also recorded. Thus, social inequality induced both an aversive autonomic response (SIH) and an approaching behavior, potentially indicating satisfaction. The sight of mice engaged in eating behavior has informative value regarding the availability of food resources for the non-eating mice, leading to interest and approach behavior despite the fact that the sight itself induces stress.

Observing conspecifics in pain constitutes another type of inequality paradigm. Approach behavior towards conspecifics in pain may have several explanations. Watanabe (2012) reported that mice spent more time in the compartment where there was a cage mate injected with a small amount of low-dose formalin, an irritant compound, in its paw formalin compared to the time spent in the compartment with an intact cage mate. Langford et al. (2010) also reported that mice placed in the presence of a cage mate confined in a container spent more time with the cage mate if it was in pain than if it was without pain. Thus, the injured cage mate had a satisfactory effect on the subjects according to Thorndike’s definition. However, approaching a cage mate may also indicate a kind of rescue behavior, information-seeking behavior about possible danger, or even, although less likely, schadenfreude (a rewarding effect deriving from the observation of the conspecific in pain). Approaching an injured conspecific may also be dangerous because of possible infection. In fact, after mice are primed with cadaverine, a compound with the odor of decomposed animal tissues, they have been shown to avoid conspecifics that exhibit sickness behavior (Renault et al., 2008). Approach behavior could also be a response to the ultrasonic vocalizations of the mouse in pain (Ko et al., 2005). In addition, subordinate mice were shown to approach dominant mates in pain more than non-dominant mates without pain, whereas dominant mice did not approach subordinate mates in pain (Watanabe, 2012). Hence, social rank order affects approaching behavior to conspecifics in pain. Interestingly, human schadenfreude is also sensitive to social rankings (Feather, 2008).

Although mice in the aforementioned study were shown to approach the formalin-injected cage mate, a conditioned place preference test with formalin-injected mates found conditioned aversion to the compartment associated with the formalin-injected mate (Watanabe, 2012). Therefore, the choice preference and conditioned place preference tests yielded contradictory results. A possible explanation could be that in the conditioned place preference, the compartment previously associated with an injured cage mate no longer held informative value (hence losing the possibility to attract the interest of the test mice), while the memory of the aversive value of the event would still be present, leading to conditioned place aversion. However, further studies are required to clarify this possibility. Combining the recording of physiological indices with behavioral testing provides a new window for studying social inequality aversion, and thermography is a promising tool for such studies.

## 5. Thermography in other animals

Infrared thermography has been used to study emotional responses in a wide variety of animals, including macaques, chimpanzees, marmosets, dogs, cats, rabbits, pigs, horses, cattle. and sheep (Travain and Valsecchi, 2021). The advantages of thermography, namely the simultaneous recording of multiple individual animals without disturbing their behavior, make it a valuable new tool for animal research. Indeed, a promising area of research is the recording of wild animals. For instance, Heintz et al. (2019) measured the temperatures of wild chimpanzees in Budongo Forest, Uganda, when exposed to vocalizations of conspecifics. Another promising area of research is animal welfare of farm animals (Mota-Rojas et al., 2021), as demonstrated by a study conducted by Cannas et al. (2018) who used this method to measure freely moving sheep. As previously mentioned, although infrared thermography measurements are angle-sensitive, the post-hoc selection of recorded data enables to provide reliable results.

## 6. New directions: potential of infrared thermography as an animal-friendly method to study rodent cognition and emotion

Psychological arousal can be divided into cognitive arousal (associated with cognitive processing and attention) and affective arousal (associated with emotional experience). We have shown, in the previous paragraphs, that infrared imaging can detect changes in the level of psychological stress, which is a type of affective arousal.

Although the DMH-BAT system is a thermoregulation system driven by stress, alternative mechanisms, as increase of heart beat and blood pressure, can also lead to increase of body temperature, and infrared thermography could hence be used to evaluate also psychological arousal states different from stress in future studies. Mice are neophilic, i.e., they are naturally attracted by novelty. Cognitive arousal can be elicited in mice by presentation of a novel stimuli vs. a familiar stimulus. Affective arousal, on the other hand, can be elicited by delivery of a reward or other emotionally salient stimuli.

It would be interesting to test infrared thermography in cognitive and affective essays unrelated to stress induction and verify if this technique is able to detect significant thermal changes during interaction with the test stimulus in comparison to interaction with the control stimulus. Several behavioral tests would be suitable for this type of comparison. Regarding cognitive arousal, thermal imaging could be used to measure thermal changes during exploration of novel vs. familiar objects in the novel object recognition test (d’Isa et al., 2014) or during exploration of novel vs. familiar environments in the hole-board test (d’Isa et al., 2021). Concerning affective arousal, infrared thermography could be employed in tests for reactivity to rewards, in the social preference test (Lammert et al., 2018; Gu et al., 2022), in the sexual preference test (Linnenbrink and von Merten, 2017; Nomoto et al., 2018; Guarraci and Frohardt, 2019) or even in social communication studies, for example to evaluate reactivity to conspecific ultrasonic vocalizations in the two-choice vocalization playback test (Asaba et al., 2015).

Researchers have recently highlighted the importance of employing animal-friendly tests in behavioral neuroscience, underlining how these tests would improve both animal welfare and validity of scientific results (Voikar and Gaburro, 2020; d’Isa and Gerlai, 2023). Indeed, infrared thermography could be a very useful tool for animal-friendly studies of cognition and emotion in rodents, which makes it a particularly promising method for behavioral neuroscience.

## Author contributions

SW conceived, wrote, and revised the manuscript and created the figures.
