# Erratum: Infrared thermography for non-invasive measurement of social inequality aversion in rodents and potential usefulness for future animal-friendly studies

**DOI:** 10.3389/fnbeh.2023.1192617

**Published:** 2023-03-31

**Authors:** Frontiers Production Office

**Affiliations:** Frontiers Media SA, Lausanne, Switzerland

**Keywords:** infrared thermography, stress-induced hyperthermia, inequality aversion, advantageous inequality, disadvantageous inequality

Due to a production error, the headings for each section were incorrectly numbered under the heading **1. Introduction**. The heading **1. Introduction** has been removed. The section numbers have been corrected as follows:

1.1. The mechanism of infrared thermography to 1. The mechanism of infrared thermography

1.2. Analysis of social inequality aversion by stress-induced hyperthermia to 2. Analysis of social inequality aversion by stress-induced hyperthermia

1.3. Social inequality aversion in rodents to 3. Social inequality aversion in rodents

1.3.1. Social inequality in restraint stress to 3.1. Social inequality in restraint stress

1.3.2. Social inequality in food delivery to 3.2. Social inequality in food delivery

1.4. Contradiction between behavioral preference and autonomic response to 4. Contradiction between behavioral preference and autonomic response

1.5. Thermography in other animals to 5. Thermography in other animals

1.6. New directions: potential of infrared thermography as an animal-friendly method to study rodent cognition and emotion to 6. New directions: potential of infrared thermography as an animal-friendly method to study rodent cognition and emotion

The publisher apologizes for this mistake. The original article has been updated.

